# Supplementary material for: Identification of photoperiod-induced specific miRNAs in the adrenal glands of Sunite sheep (Ovis aries)
Source: Front Vet Sci. 2022 Jul 22;9:888207. doi: 10.3389/fvets.2022.888207 (PMC9354845; doi:10.3389/fvets.2022.888207)
Supplement: Supplementary file 1 [file Data_Sheet_1.ZIP › Supplementary file/miRDeep2 file.zip!/miRDeep2 file/Know_Structure/SP42A2/oar-mir-433.pdf]

miRBase precursor : oar-mir-433
Total read count : 2040
oar-miR-433-5p read count : 1
oar-miR-433-3p read count : 2039
remaining reads : 0

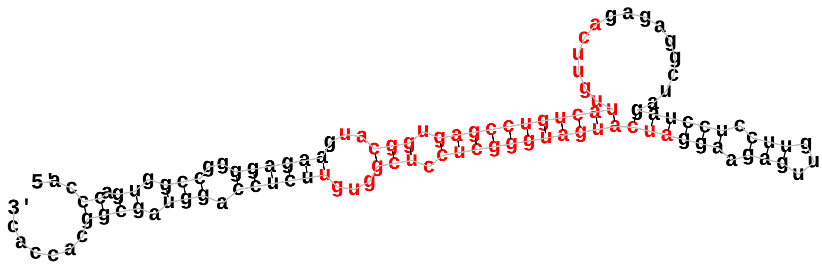

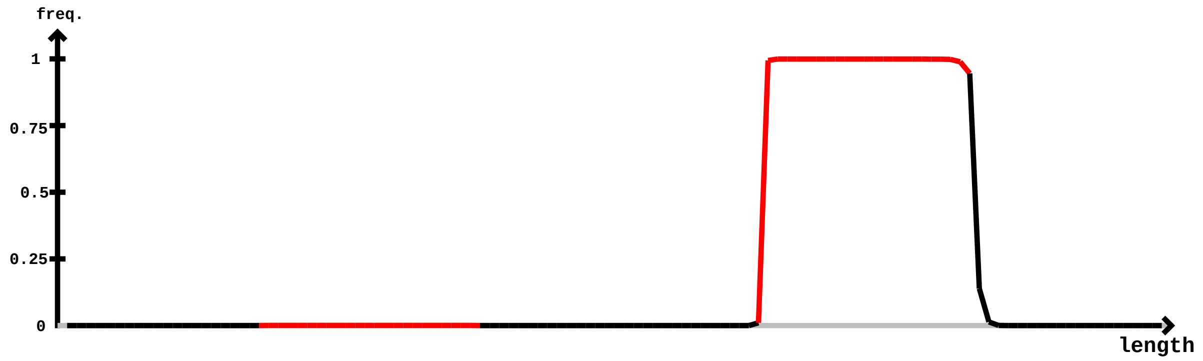

oar-miR-433-5p

oar-miR-433-3p

5'- acccaguggccggggagaaguacggugagccugucauuguucagagaggcuagauccuccuuguugagaaggaucaugaugggcuccucgguguucuccagguagcggcaccac -3'	exp		
..((.((.(((..((((((...(((.(((((((((((...............((((((.(((...))).))))))))))))))))).)))...)))))).))).))))......	reads	mm	sample
....................uacggugagccugucauuguuc..	1	0	seq
....gaucaugaugggcuccucggu......................	1	0	seq
....gaucaugaugggcuccucggug.....................	3	0	seq
....gaucaugaugggcuccucggugu....................	13	0	seq
....gaucaugaugggcuccucgguguu...................	4	0	seq
....aucaugaugggcuccuc.........................	1	0	seq
....aucaugaugggcuccucgg.......................	2	0	seq
....aucaugaugggcuccucggu......................	17	0	seq
....aucaugaugggcuccucggug.....................	85	0	seq
....aucaugaugggcuccucggugu....................	1626	0	seq
....aucaugaugggcuccucgguguu...................	248	0	seq
....aucaugaugggcuccucgguguuc..................	27	0	seq
....aucaugaugggcuccucgguguucu.................	1	0	seq
....ucaugaugggcuccucggug.....................	1	0	seq
....ucaugaugggcuccucggugu....................	7	0	seq
....ucaugaugggcuccucgguguu...................	3	0	seq
